# Editorial: The human microbiome: A new frontier in personalized cancer therapy

**DOI:** 10.3389/fcimb.2022.1028120

**Published:** 2022-09-14

**Authors:** Valeriy Poroyko, Edwin Ramos Manuel, Elena Ilina

**Affiliations:** ^1^ Microbiology Department, LabCorp Drug Development, Indianapolis, IN, United States; ^2^ Beckman Research Institute, City of Hope, Duarte, CA, United States; ^3^ Laboratory of Molecular Genetics of Microorganisms, Federal Research and Clinical Center of Physical-Chemical Medicine, Moscow, Russia

**Keywords:** cancer, microbiome, personalized therapy, metaorganism, bioavailability

The statistics from the National Cancer Institute show that cancer remains among the leading causes of mortality worldwide. It also indicates that the population burden of the disease continues to grow. Gene- and immuno-therapies, combined with extensive molecular testing, have successfully dealt with the inherent molecular variability of cancer lesions personifying modern cancer treatment. The best indicator of progress, accredited to precision and personalized therapy, is the change in age-adjusted mortality rates that declined steadily between 2006 and 2015. However, the treatment outcomes still vary, suggesting that ‘personalized’ cancer therapy is yet to become fully personalized. Improving outcomes demands searching for the confounding factors still impacting the treatment.

Since the initiation of the Human Microbiome Project, humans have been recognized as ‘meta organisms’ due to their close symbiotic relationship with their intestinal microbiota. The recent discovery of tumor-associated bacterial dwellers revealed an even higher degree of interaction between microbiota and the human host, accentuating the spatial proximity of bacterial cells to the very target of targeted cancer therapy. This Research Topic explores microbiota’s role in a tumor-bearing meta-organism and how it may affect personalized cancer treatment further.

The work of Shi et al., “*Characteristics and Clinical Implications of the Nasal Microbiota in Extranodal NK/T-Cell Lymphoma, Nasal Type*,” discusses the impact of nasal cavity cancer on the structure of the microbial population. The study of nasal microbiota of patients with Natural killer/T cell lymphoma (NKTCL), chronic rhinosinusitis (CRS), and matched healthy controls revealed a unique structure of the nasal microbiota attributed to NKTCL patients. The authors demonstrated the depletion of *Corynebacterium* in the nasal microbial communities of CRS and NKTCL patients, while the abundance of *Staphylococcus* was highest in the NKTCL group. Based on a panel of taxa, they can discern NKTCL and CRS patients with an excellent classification power (AUC of 0.875). The study not only points out that the diagnostic panel of microbiota in NKTCL contains a set of promising biomarkers but suggests that the nasal microenvironment is different between NKTCL and CRS. The manuscript exposes certain species as having a selective advantage in the cancer-related environment that may contribute to the differences in the pathogenesis of these malignancies.

On a similar note, the study of Gao et al., *“Alterations of Gut Mycobiota Profiles in Adenoma and Colorectal Cancer,”* uses metagenomic sequencing to study the gut mycobiome of patients with colonic adenoma and colorectal cancer. The matching healthy control group was used as a control. Despite the scarcity of gut fungal communities, accounting for 0.1% of total gastrointestinal microorganisms, the authors found that the fungal species were suitable as biomarkers of colorectal cancer. They were able to build a predictive model using a fungal species panel. Together with their previous study, Gao *et al*. reveal the tremendous effect cancer has on the body’s habitats, which is reflected in changes in the host microbiome. The question remains whether these changes affect cancer pathogenesis and susceptibility to treatment.

The manuscript of Genton et al. *“Metataxonomic and Metabolic Impact of Fecal Microbiota Transplantation from Patients with Pancreatic Cancer into Germ-Free Mice: A Pilot Study”* raises the question of whether cancer symptoms can be attributed to the impact of disease-related alterations in the gut microbiome. To establish causality, the authors performed a fecal material transplant (FMT) from pancreatic cancer (PC) patients to a colony of gnotobiotic mice. They found that PC FMT is associated with decreased visceral fat accumulation among the recipient mice compared to the healthy individuals’ FMT recipient group. Therefore, the authors exposed a phenomenon of Body Weight Loss, the prevalent symptom in patients with pancreatic cancer, as causally associated with cancer-related gut microbiota. The findings warrant future investigation to confirm whether the altered microbial communities secrete metabolic and immune-altering elements to cause these outcomes.

The review by Yue et al., *“Microbiota-Host-Irinotecan Axis: A New Insight Toward Irinotecan Chemotherapy,”* provides an example of a comprehensive study dissecting the Microbiota-Host-Drug axis while discussing the relationship between gut microbiota and the drug Irinotecan (CPT11), a cytotoxic agent affecting the fast-growing cells of the tumor, epithelial cells, and commensal bacteria. The authors discuss how the microbiota influences the efficacy and toxicity of CPT11 chemotherapy through several mechanisms: microbial ecocline, catalysis of microbial enzymes, and immunoregulation. They emphasize several interventions such as bacterial β-glucuronidase-specific inhibitors, dietary interventions, probiotics, and strain-engineered interventions as emergent microbiota-targeting strategies capable of improving CPT11 chemotherapy efficiency and alleviating toxicity. The manuscript provides a model framework for researchers viewing microbiota manipulation as a feasible approach to personalize cancer therapy.

The host-dependent variations have yet to be fully shown in anticancer drug–microbiota interactions. The study of Hobson et al., *“A Microbiota-Dependent Response to Anticancer Treatment in an In Vitro Human Microbiota Model: A Pilot Study with Hydroxycarbamide and Daunorubicin,”* provides the tools for such investigation. The authors propose an *in vitro* human microbiota model based on the MiniBioReactors Array (MBRA). MBRA allows a dynamic, stable anaerobic culture of human-derived microbiota to study anticancer drug–microbiota interactions. The proposed simulator helps in predicting the effects elicited along the Microbiota-Host-Drug axis. In the model study, the authors analyze how the two drugs Daunorubicin and hydroxycarbamide impact the model gut microbiota. The manuscript reports that Daunorubicin affects alpha- and beta-diversity as well as a proverbial ratio of Firmicutes/Bacteroidetes in a donor-dependent manner. At the same time, the impact of hydroxycarbamide on microbiota composition was insignificant and was not donor-dependent. These data are crucial to objectify the donor-dependent effect of a drug molecule and the molecule-dependent influence of the drugs, excluding the primary confounding bias of the host and its environment, such as diet. The disparities in the magnitude of effects between donors should be further explored to understand the mechanisms underlying drug-associated dysbiosis. These elements confirm the importance of personalized medicine, for which the MBRA model seems particularly valuable.

Both parts of the tumor-bearing metaorganism may be affected by the tumor and are equally capable of responding to anticancer treatment. The current paradigm of personalized medicine accounts for a human host’s unique genetic and physiological background but ignores its microbial counterpart. The microbiota can modify its structure in response to tumor development and treatment, simultaneously affecting the bioavailability of the administered drug and the host immunological milieu. The notion of these microbiota-related alterations challenges the rigid framework of the current cancer treatment routines. It demands microbiome analysis to become part of the decision-making process while developing a personalized treatment strategy. Ultimately, state-of-the-art microbial ecology can be instrumental in providing analytical support to physicians to advance the efficacy of cancer treatment.

## Author contributions

VP - writing the manuscript; EM and EI - manuscript editing and approval.

## Conflict of interest

Author V. Poroyko was employed by the company “LabCorp Drug Development”.

The remaining authors declare that the research was conducted in the absence of any commercial or financial relationships that could be construed as a potential conflict of interest.

## Publisher’s note

All claims expressed in this article are solely those of the authors and do not necessarily represent those of their affiliated organizations, or those of the publisher, the editors and the reviewers. Any product that may be evaluated in this article, or claim that may be made by its manufacturer, is not guaranteed or endorsed by the publisher.

